# Effect of Formulation and Processing Variables on the Characteristics of Tolmetin Microspheres Prepared by Double Emulsion Solvent Diffusion Method

**DOI:** 10.4103/0250-474X.62251

**Published:** 2010

**Authors:** M. Jelvehgari, A. Nokhodchi, M. Rezapour, H. Valizadeh

**Affiliations:** Department of Pharmaceutics, Faculty of Pharmacy, Tabriz University of Medical Sciences, Tabriz, Iran; 1 Drug Applied Research Center, Tabriz University of Medical Sciences, Tabriz, Iran; 2 Medway School of Pharmacy, Universities of Kent and Greenwich, Central Ave., Chatham Maritime, ME4 4TB, Kent, UK; 3 Biotechnology Research Center, Tabriz University of Medical Sciences, Tabriz, Iran; 4 Research Center for Pharmaceutical Nanotechnology, Tabriz University of Medical Sciences, Tabriz, Iran

**Keywords:** Controlled release, double-emulsion, ethylcellulose, microspheres, tolmetin sodium

## Abstract

The aim of this study was to evaluate microencapsulated controlled release preparations of tolmetin sodium using ethylcellulose as a retardant material. Microspheres were prepared by using water-in-oil-in-oil (W/O_1_/O_2_) double-emulsion solvent diffusion method, using different ratios of ethylcellulose to tolmetin sodium. Span 80 was used as the droplet stabilizer and *n*-hexane was added to harden the microspheres. The prepared microspheres were characterized for their micromeritic properties, drug content, loading efficiency, production yield, and particle size. Fourier transform infrared spectroscopy, differential scanning calorimetry, X-ray powder diffractometry and scanning electron microscopy were used to characterize microparticles. The *in vitro* release studies were performed in pH 1.2 and 7.4. The prepared microspheres were spherical in shape. The drug-loaded microspheres showed near to the theoretical of entrapment and release was extended up to 24. The X-ray diffractogram and differential scanning thermographs showed amorphous state of the drug in the microspheres. It was shown that the drug: polymer ratio, stirring rate, volume of dispersing medium and surfactant influenced the drug loading, particle size and drug release behavior of the formed microparticles. The results showed that, generally, an increase in the ratio of drug: polymer (0.5:1) resulted in a reduction in the release rate of the drug which may be attributed to the hydrophobic nature of the polymer. The *in vitro* release profile could be modified by changing various processing and formulation parameters to give a controlled release of drug from the microparticules. The release of tolmetin was influenced by the drug to polymer ratio and particle size and was found to be diffusion and erosion controlled. The best-fit release kinetic was achieved with Peppas model.

Tolmetin sodium (TOL) is a non-steroidal antiinflammatory drug (NSAID) effective in treating fever, pain and inflammation in the body[1]. Most patients benefit from tolmetin, but serious side effects can occur, which generally tend to be dose related. Therefore, it is advisable to use the lowest effective dose to minimize side effects[2]. The short plasma half-life of 30-60 min following oral dosing necessitates frequent administration of the drug in order to maintain the desired steady state levels. A popular method for the encapsulation of water-soluble drugs within water insoluble polymers is the double-emulsion solvent diffusion method. The encapsulation of TOL in Eudragit RS-100, RL-100, ethyl cellulose and poly-D, L-lactide microspheres has been described in previous works[3-5]. Moreover, ethylcellulose tolmetin loaded microspheres may show a better gastric tolerability (reduce ulcerogenic effect) than the free drug[4]. However, most of the microencapsulation techniques have been used for lipophilic drugs, since hydrophilic drugs showed low loading efficiency[6]. The present study was conducted in order to study the effects of drug-polymer ratio, stirring rate, dispersing medium and emulsifier concentrations on the incorporation efficiency, yield value, particle size and distribution, dispersed phase viscosity, surface characteristics of microspheres and dissolution characteristics. The purpose was to improve loading efficiency of water-soluble drugs and modulate release profiles.

## MATERIALS AND METHODS

Tolmetin sodium was procured from Medichem, China; ethyl cellulose 48 cP was purchased from Sigma-Aldrich, USA; Medectin^®^ was obtained from Modava, Iran, while dichloromethane, acetonitrile, Span 80, liquid paraffin, *n*-hexane, hydrochloric acid, phosphate tribasic and sodium hydroxide were from Merck, Germany. All solvents and reagents were of analytical grade.

### Preparation of microspheres:

Microspheres were prepared by using water-in oil-in oil (W/O_1_/O_2_) double emulsion solvent diffusion method with different TOL to ethylcellulose ratios (0.25:1, 0.5: 1, 0.75: 1 and 1:1). Ethyl cellulose (300 mg) and TOL (150 mg) were dissolved in 5 ml of the mixed solvent system consisting of acetonitrile and dichloromethane in a 1:1 ratio. The initial W/O emulsion was prepared by adding 2 ml of water to the drug-polymer solution while stirring using a mechanical stirrer at 500 rpm. This W/O primary emulsion was slowly added to 50 ml of light liquid paraffin, the second oil phase containing 0.5% span 80 as a surfactant while stirring by a paddle propeller at 1000 rpm, immersed in an ice water bath. After 2 h, 10 ml of *n*-hexane (non-solvent) was added to harden the microspheres and stirring was continued for a further 1 h and the hardened microspheres were collected by filtration and washed with three portions of 50 ml of *n*-hexane and air dried for 12 h.

### Determination of drug content of microspheres:

Drug amount in microspheres was determined by dissolving 10 mg of each sample in 100 ml dichloromethane. The drug concentration was determined spectrophotometrically (UV-160, Shimadzu, Japan) at 262 nm. All experiments were done in triplicate.

### Determination of loading efficiency and production yield:

The loading efficiency (%) was calculated according to the following equation, loading efficiency (%)= (actual TOL content in microparticles/theoretical TOL content)×100. The production yield of the microparticles was determined by calculating accurately the initial weight of the raw materials and the last weight of the microspheres obtained[7]. All of the experiments were performed in triplicate.

### Characterization of microspheres:

A Brookfield rotational digital viscometer DVLV-II was used to measure the viscosity (cP) of the internal and external phases at 25°. Spindle No. 1 was rotated at 100 rpm. The morphology of microparticles was examined with a scanning electron microscope (LEO 440i, England) operating at 15 kV. The samples were mounted on a metal stub with double adhesive tape and coated with platinum/palladium alloy under vacuum.

For differential scanning calorimetry (DSC) about 5 mg of sample was weighed into an aluminum pan, the pan crimped non-hermetically, and heated in the differential scanning calorimeter (DSC 60, Shimadzu, Japan) from 30 to 200° at a rate of 10° per min. X-ray diffraction analysis was performed with a (Siemens D5000, Munich, Germany) using nickel-filtered CuKα radiation (a voltage of 40 KV and a current of 20 mA). The scanning rate was 2°/min over a 2θ range of 20-60° and with an interval of 0.02°.

The infrared spectrum of the drug, microspheres containing the drug were obtained in potassium bromide discs (0.5% w/w) using a FTIR (Bomen Hartmann and Brann, Canada) spectrophotometer. A laser light scattering particle size analyzer (SALD-2101, Shimadzu, Japan) was used to determine the particle size of the drug and microparticulate formulations. Samples were suspended in distilled water (microparticles) or dichloromethane (TOL) in a 1 cm cuvette and stirred continuously during the particle size analysis.

### *In vitro* release study:

Dissolution studies were carried out using a USP basket method at 37° and 100 rpm with 750 ml of 0.1 N HCl, equilibrated at 37±0.5°. Microspheres (200 mg drug) were placed in the apparatus and 5 ml aliquots of medium were withdrawn at pre-set times over 2 h and replaced by 5 ml of fresh medium. The samples were filtered through 0.45 μm filters and used for the spectroscopic determination of the drug. Dilution with the same buffer solution was carried out if necessary. After 2 h, 250 ml of 0.2 M tribasic sodium phosphate, pre-equilibrated at 37°, were added to the dissolution vessel. The pH was immediately adjusted, if necessary, with 2N HCl or 2N NaOH to pH 6.8. Drug concentration in the samples was measured by UV spectrophotometric analysis at 315 and 322 nm for the acidic and enteric buffers, respectively. Each experiment was repeated three times.

## RESULTS AND DISCUSSION

**Fig. 1:**
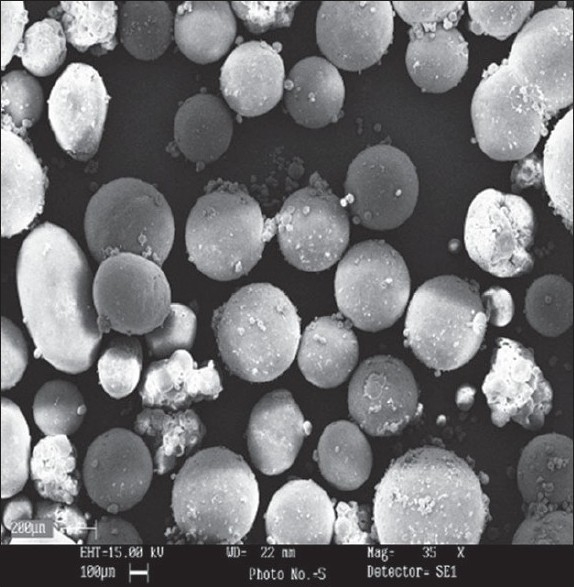
SEM of a spherical microspheres F_2_ (drug:polymer ratio, 0.5:1), *magnification= 35X

Microspheres were formed after a series of steps like solvent extraction and solvent evaporation and addition of non-solvent. Acetonitrile is a unique organic solvent which is polar, water miscible and oil immiscible. All other polar solvents are oil-miscible and do not form emulsions of the polymer solution in oil[8]. Dichloromethane is non-polar and oil miscible. Using acetonitrile alone as a solvent did not ensure formation of a stable emulsion, and non-polar solvent such as dichloromethane was included to decrease polarity of the acetonitrile solution[8]. Therefore, during the formation of microspheres, dichloromethane is extracted by liquid paraffin and acetonitrile is evaporated during stirring. One method of ensuring high entrapment efficiency of water-soluble active ingredients is to use a hydrophobic processing medium into which the hydrophobic macromolecule is unlikely to migrate out. Microspheres were prepared using different drug-polymer ratios (0.25:1, 0.5: 1, 0.75: 1 and 1: 1) as shown in Table 1. The drug-polymer ratio was varied by maintaining the amounts of polymer and solvent constant in all preparations, and changing the amount of drug. The results of the effect of drug-polymer ratio on production yield, drug loading efficiency and mean particle size are shown in Table 1. The pore formation is induced by diffusion of solvent from surface of the microparticles. In all of the formulations, the mean amount of drug entrapped in prepared microspheres was near to the theoretical value, since the drug loading efficiency is almost 100%. The encapsulation efficiency of the drug depended on the solubility of the drug in the solvent and continuous phase. Using higher amounts of the drug caused a slight increase is viscosity of dispersed phase. Entrapment efficiency of polypeptides was increased by enhancing the viscosity builders[9]. Generally, increasing the drug-polymer ratio increased the production yield, when the ratio of drug-polymer increased from 0.25:1 to 1:1 the production yield was decreased (p<0.05). The reason for decreased production yield at high drug: polymer ratios could be due to decreased diffusion rate of solvents (acetonitrile and dichloromethane 1:1) from concentrated solutions into initial emulsion. Size of microspheres was found to be increased with the increase in the concentration of drug (Table 1). It can be attributed to the fact that with the higher diffusion rate of non-solvent to polymer solution the smaller size of microcapsules is easily obtained[10]. A volume-based size distribution of drug, polymer, and drug loaded microspheres indicated a log-probability distribution. Mean particle size of original tolmetin and ethylcellulose was 51.21±0.47 μm and 76.09±0.33 μm, respectively. SEM of microspheres (as F_2_) was demonstrated in fig. 1. In fact viscosity of dispersed phase was increased from F_1_ (0.25:1) to F_4_ (1:1). The results showed that the apparent viscosities of the different drug: polymer ratios (0.25:1, 0.5:1, 0.75:1, 1:10) were 14, 23, 34 and 43.3 mPa.S, respectively.

**TABLE 1:** EFFECT OF DIFFERENT PROCESSING VARIABLES ON CHARACTERISTICS OF TOLMETIN SODIUM MICROPARTICLES

parameter	Formulation	Process variable	Production yield (%±SD)	Theoretical drug content (%)	Mean drug entrapped (%±SD)	Mean particle size (μm±SD)	Drug loading efficiency (%±SD)
Drug to polymer ratio	F_1_	0.25:1	58.6±4.24	20	19.7±3.21	189.90±0.21	98.35±3.35
	F_2_^a^	0.5:1	40±1.24	33.33	33.1±3.56	209.02±0.19	99.40±6.31
	F_3_	0.75:1	28.6±3.58	42.85	45.1±5.65**	279.30±0.20	105.32±1.32
	F_4_	1:1	23.3±3.13	50	51.5±3.15	401.01±0.16	103.06±3.15
Stirring rate (rpm)	F_2_-1	500	48.8±3.80	33.33	35.9±3.75	470.05±0.18	107.78±6.25
	F_2_-2^a^	1000	40.0±1.24	33.33	33.3±3.56	209.02±0.20	99.40±6.31
	F_2_-3	2000	55.6±6.19	33.33	34.7±3.46	113.53±0.17	104.32±7.23
Emulsifier concentration %	F_2_-4^a^	0.5	40±1.24	33.33	33.1±3.56	209.02±0.90	99.40±6.31
	F_2_-5	1	78.9±7.68	33.33	32.8±4.47	198.98±0.19	98.35±5.24
	F_2_-6	2	43.3±6.59	33.33	32.3±2.33	190.30±0.11	97.06±3.56
Volume of dispersing medium (ml)	F_2_-7^a^	50	40±1.24	33.33	33.1±3.56	209.02±0.20	99.40±6.31
	F_2_-8	100	50±4.58	33.33	27.2±5.51	140.10±0.20	81.74±4.65**
	F_2_-9	200	55±6.35	33.33	17.1±0.21	135.77±0.15	51.23±2.35

a F_2_ is selected formulation and process variable was performed on it. F_2_-2, F_2_-4 and F_2_-7 are the same as F_2_ formulation

The effect of stirring rate on the physical characteristics of the microspheres was examined for formulation F_2_. The results of stirring rate on the mean particle size diameter of microspheres, drug entrapment and production yield are listed in Table 1. The results showed that increasing the stirring rate from 500-1500 rpm did not affect the production yield and the drug content (p>0.05). Tolmetin sodium is water soluble with less affinity to distribute from internal phase of initial emulsion to oily phase (outer phase in second emulsion). Therefore, no reduction in drug content was seen in comparison to the theoretical drug content. Table 1 also shows that the stirring rate employed had effect on particle size diameter. At stirrer speed of 1500 rpm (F_2_-3), the resulting high turbulence, caused frothing and adhesion to the container wall. Therefore, the mean particle size of microspheres decreased. The desired spherical and not aggregated microspheres were obtained at stirring speeds of 1000 rpm (F_2_-2, Table 1). Any increase in mean particle size at lower stirring rate as 500 rpm (F_2_-1) can be attributed to increased tendency of globules to coalescence and aggregates.

When 0.25% span 80 was incorporated, microspheres were not formed because the low emulsifier content failed to prevent droplet coalescence in the oil medium; as a result mean particle size was increased. The type and concentration of emulsifier has a key role to play in the preparation of microspheres[11]. According Table 1, when emulsifier concentration was increased, size of microcapsules F_2_-5 and F_2_-6 (containing 1 and 2% emulsifier, respectively) were smaller than F_2_-4, also at F_2_-6 sphericity of microparticles was decreased and production yield increased (p<0.05). Span 80 was used to stabilize the secondary emulsification process and have a high disparity for the present emulsion system by reducing the surface tension at the interface. The mean particle size decreased with increasing amount of emulsifier (Table 1). This is probably a consequence of stabilization of the oil droplets with Span 80. Spherical microspheres were formed when the Span 80 content was at 0.5%. The *n*-hexane, non-solvent for the polymer added at this stage may lead to a quick precipitation of the polymer leaving the surface of microspheres porous.

The volume of processing medium (outer phase, O_2_) significantly influenced the entrapment efficiency of the microspheres (Table 1). As the volume of processing medium was increased from 100 ml to 200 ml, the entrapment efficiency significantly decreased from 32% to 17% (comparing F_2_-7 and F_2_-9) (p<0.05). As the volume of processing medium was increased, the emulsion droplets probably moved freely in the medium, thus reducing collision induced aggregation and yielding small and uniform microspheres. This could also be the reason for higher drug extraction into the processing medium resulting in lower entrapment efficiency.

**Fig. 2:**
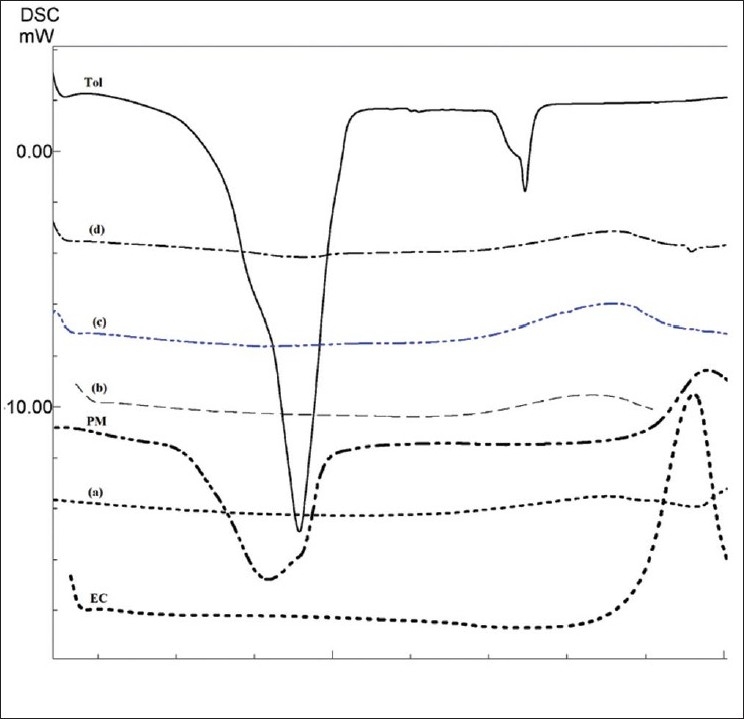
DSC thermogram of different preparations Ethylcellulose (EC), F_1_, 0.25:1 (a), physical mixture PM), F_2_, 0.5:1 (b), F_3_, 0.75:1 (c), F_4_, 1:1 (d), and tolmetin (TOL).

**Fig. 3:**
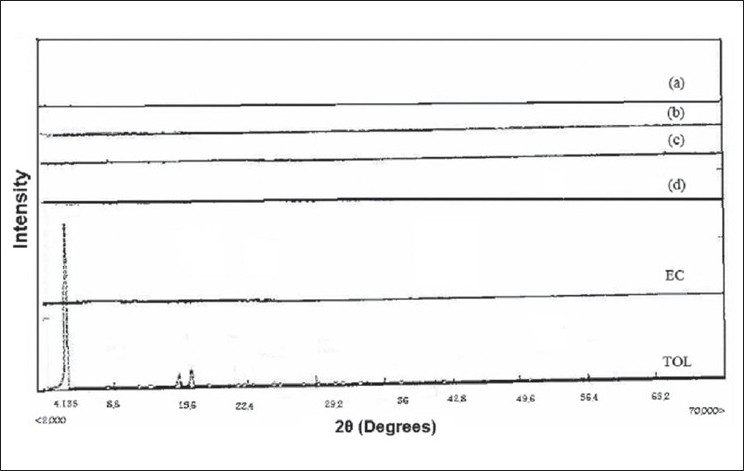
X-ray diffraction pattern of different formulations F_1_, 0.25:1 (a); F_2_, 0.5:1 (b); F_3_, 0.75:1 (c); F_4_, 1:1 (d); ethylcellulose (EC); TOL (tolmetin).

The drug may have been dispersed in crystalline or amorphous form or dissolved in the polymeric matrix during formation of the microspheres. Any abrupt or drastic change in the thermal behavior of rather the drug or polymer may indicate a possible drug-polymer interaction[12]. The endothermic peak of pure drug was observed at about 160° (fig. 2). However in the thermogram of the microparticles there was no endothermic peak of the drug melting, suggesting the amorphous state of the drug in the microparticles. The X-ray diffraction patterns of pure drug, shows that the pure drug is crystalline in nature (fig. 3). However when it was incorporated into the polymer matrix, the principal peaks of the drug was disappeared. This could be ascribed to the amorphous state of the drug in the microparticles. This confirms the results obtained from DSC experiments.

**Fig. 4:**
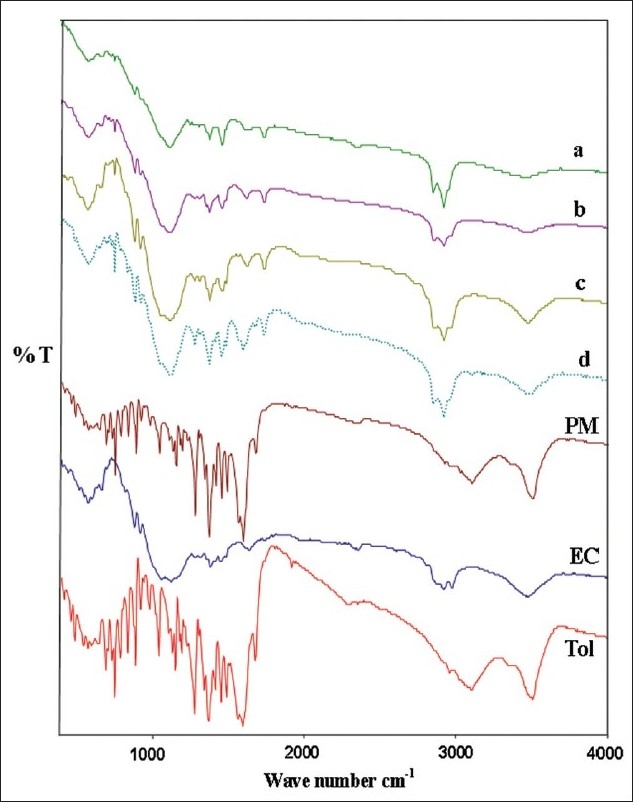
FTIR spectrum of different formulations Tolmetin (Tol); ethylcellulose (EC); F_1_, 0.25:1 (a); F_2_, 0.5:1 (b); F_3_, 0.75:1 (c); F_4_, 1:1(d); and physical mixture (PM).

As shown in fig. 4, there was no significant difference in the FT-IR spectra of physical mixture and drug-loaded microspheres. The characteristic OH stretching, NH stretching, C-H stretching and C=O stretching of pure drug was unchanged in the spectra of the microspheres. The results suggest the stability of the drug during the encapsulation process.

**Fig. 5:**
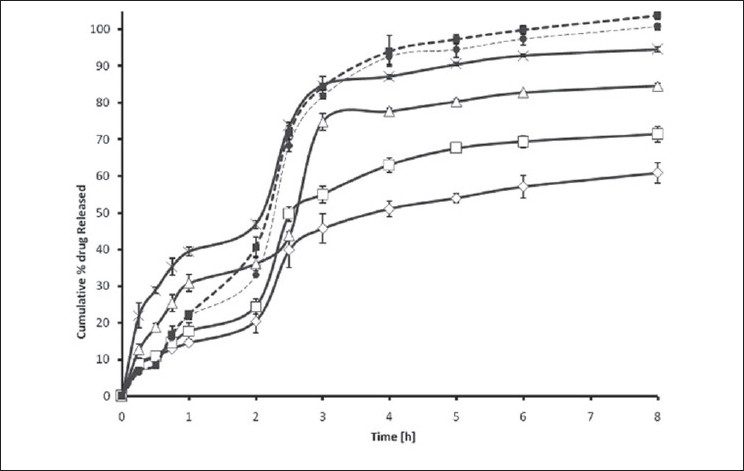
Cumulative percent release of tolmetin sodium from microspheres Cumulative percent release to tolmetin sodium from microspheres prepared with different polymerto dug ratio. F_1_, 0.25:1 (-◊-), F2, 0.5:1 (-□-), F3, 0.75:1 (-Δ-), F4, 1:1 (-×-), Physical mixture (--■-), Medectin^®^ (--•-). Each data point represents mean±SD (*n*=3).

The *in vitro* release of TOL from ethyl cellulose microspheres exhibited initial burst effect, which may be due to the presence of some drug particles on the surface of the microspheres. The initial burst effect may be attributed as a desired effect to ensure initial therapeutic plasma concentrations of drug. The release profiles are illustrated in fig. 5. Due to its weakly acidic nature, TOL always shows an expected increase in dissolution after the change of pH from 1.2 to 6.8. However, with respect to the physical mixture, microparticles showed slighter modification of dissolution profile in pH 6.8. For microparticles, dissolution of TOL at pH 1.2 was strongly reduced and the initial burst effect at pH 6.8 was moderated, resulting in an overall slower drug release. In most cases, a biphasic dissolution profile was observed at pH 6.8: the initial rapid drug leakage generally ended very early (within first 30-60 min after the change of dissolution medium pH to 6.8); for the remaining time, nearly linear behavior was observed. After such a phase, two phenomena can combine in enhancing in the diffusion of the remaining dispersed drug into the bulk phase as well as the formation of pores within the matrix due to the initial drug dissolution; particle wetting and swelling which enhances the permeability of the polymer to the drug[13] (fig. 5). The results indicated that factors such as polymer-drug ratio, stirring speed, surfactant concentration in secondary emulsification and volume of processing medium of secondary emulsification govern the drug release from these microspheres. Drug release rates increased with increasing amounts of TOL in the formulation. Higher level of TOL corresponding to lower level of the polymer in the formulation resulted in an increase in the drug release rate. As more drugs are released from the microspheres, more channels are probably produced, contributing to faster drug release rates. However, fig. 5 shows that the burst effect is higher when the TOL to polymer ratio is 1:1 (F_4_) and 0.75:1 (F_3_). Moreover, almost the same amount is released at 8h from the tablet and F_4_. Therefore formulations F_4_ and F_3_ could not prolong the release of TOL. Only formulations F_1_ and F_2_ are prolonged release, which could be due to the thicker polymer membrane that controls the release rate. Statistical analysis of data was performed by comparing the dissolution efficiency (DE), t_50%_ (dissolution time for 50% fractions of drug); the “difference factor, f_1_” and “similarity factor, f_2_” (used to compare multipoint dissolution profiles)[14] (Table 2). DE was calculated from the area under the dissolution curve at time and expressed as percentage of the area of the rectangle described by 100% dissolution in the same time. F_1_, F_2_ microspheres showed lower dissolution efficiency 56.20 and 65.60% respectively and slow dissolution. Medectin^®^ tablet and physical mixture had higher release in comparison with microspheres (p<0.05, Table 2 and fig. 5).

**TABLE 2:** COMPARISON OF VARIOUS RELEASE CHARACTERISTICS FROM DIFFERENT FORMULATIONS

Formulation	at 50% (h)	bDE	cQ2 (mg)	dQ8 (mg)	Difference factor	Similarity factor
F1	4	56.2	20.41±3.15	60.92±2.72	37	31.65
F2	3	65.06	24.36±2.04	71.39 ±2.06	25.97	40.4
F3	3	81.43	36.08±1.99	84.61±0.66	17.16	48.07
F4	2.5	88.57	46.75±1.10	94.41±0.61	19.78	43.62
Physical mixture	2.5	95	40.60±2.88	103.89±1.06	13.76	53.6
Medectin^®^	2.5	82.79	38.10±1.52	60.41±1.88	0	100

The change of stirring speed of the secondary emulsification process also influenced the drug release profile. As the concentration of Span 80 increased a faster drug release was observed. This may be attributed to the presence of greater amount of free drug on the surface of the microspheres with increasing the concentration of Span 80 used for secondary emulsification process. There is no differences on the drug released from the microspheres at pH=1.2. The faster drug release was observed from microspheres prepared using large volume of processing medium at pH =6.8 also this formulation had less drug entrapment (F_2_-9). It may be due to the higher migration of drug to the surface of the microspheres during solvent evaporation from the freely moved emulsion droplets in large volume of processing medium.

The *in vitro* release profiles were fitted on various kinetic models in order to find out the mechanism of drug release (Table 3)[15]. The fit parameters to Higuchi, first-order, Peppas and zero-order equations. The rate constants were calculated from the slope of the respective plots. High correlation was observed for the Peppas model. The data obtained were also put in Korsemeyer-Peppas model in order to find out n value, which describes the drug release mechanism. The n value of microspheres of different drug to polymer ratio was between 0.51-0.91, indicating that the mechanism of the drug release were diffusion and erosion controlled.

**TABLE 3:** FITTING PARAMETERS OF THE *IN VITRO* RELEASE DATA TO VARIOUS RELEASE KINETICS MODELS

Order		F1	F2	F3	F4	Medectin^®^	Physical Mixture
K_0_	Zero	0.0004	0.0004	0.0005	0.0005	0.0006	0.00013
RSQ		0.457	0.4078	0.493	0.3874	0.3543	0.7848
D(SS)%		919.318	933.787	943.654	1009.95	881.477	649.399
K_1_	First	0.0007	0.0009	0.0023	0.0024	0.0018	0.0072
RSQ		0.5716	0.4744	0.8541	0.65	0.4896	0.892
D(SS) %		781.9	771.256	524.534	625.915	488.386	389.424
b	Peppas	0.6658	0.7665	0.519	0.3724	0.9178	0.9155
K_p_		0.0115	0.0087	0.0329	0.0823	0.0019	0.005
RSQ		0.9453	0.9326	0.9722	0.9816	0.9674	0.9524
D(SS) %		163.505	136.341	35.344	16.625	66.127	57.58
K_h_	Higuchi	0.0201	0.0231	0.0278	0.0252	0.0315	0.0732
RSQ		0.7399	0.6699	0.7412	0.6524	0.6176	0.9455
D(SS) %		460.555	286.844	335.631	567.681	818.665	1261.28

*Kinetics models equations (Zero order: f=kt, First order: Ln(1-f)=kt, Peppas: Ln(f)=ktb, Higuchi: f=kt 0.5, % D(ss)= percent error, RSQ= Regression coefficient

In conclusion, tolmetin sodium microspheres were prepared using double emulsion (W/O_1_/O_2_) solvent diffusion method. Drug: polymer ratio, stirring speed, emulsifier and dispersing medium influenced the sphericity of the microspheres. The entrapment efficiency was high for all formulations. The encapsulation efficiency was less influenced with changing the stirring speed of the second emulsification process, emulsifier concentration and dispersing medium concentration.

It was observed that at higher drug concentration, the mean particle size of the microspheres is high but increasing the stirring speed and emulsifier content, resulted in smaller mean particle size of microspheres. The assessment of the release kinetics revealed that drug release from tolmetin microspheres followed Peppas model. It was suggested that mechanism of drug release from microspheres was diffusion and erosion controlled.
